# Triboelectric‐Inertial Sensing Glove Enhanced by Charge‐Retained Strategy for Human‐Machine Interaction

**DOI:** 10.1002/advs.202408689

**Published:** 2024-11-22

**Authors:** Bo Yang, Jia Cheng, Xuecheng Qu, Yuning Song, Lifa Yang, Junyao Shen, Ziqian Bai, Linhong Ji

**Affiliations:** ^1^ State Key Laboratory of Tribology in Advanced Equipment Department of Mechanical Engineering Tsinghua University Beijing 100084 China; ^2^ Beijing Lvkedu Science and Technology Co. Ltd. Beijing 100190 China; ^3^ School of Engineering and Technology China University of Geosciences (Beijing) Beijing 100083 China

**Keywords:** artificial intelligence, gesture recognition, human‐machine interaction (HMI), signal processing, smart glove, triboelectric sensor

## Abstract

As technology advances, human‐machine interaction (HMI) demands more intuitive and natural methods. To meet this demand, smart gloves, capable of capturing intricate hand movements, are emerging as vital HMI tools. Moreover, triboelectric‐based sensors, with their self‐powered, cost‐effective, and material various characteristics, can offer promising solutions for enhancing existing glove systems. However, a key limitation of these sensors is that charge leakage in the measurement circuit results in capturing only transient signals, rather than continuous changes. To address this issue, a charge‐retained circuit that effectively prevents triboelectric signal attenuation is developed, enabling accurate measurement of continuous finger movements. This innovation forms the foundation of a highly integrated smart glove system, enhancing HMI functionality by combining continuous triboelectric signals with inertial sensor data. The system showcases a diverse range of applications, including complex robotic control, virtual reality interaction, smart home lighting adjustments, and intuitive interface operations. Furthermore, by leveraging artificial intelligence (AI) techniques, the system achieves accurate recognition of complex sign language with an impressive 99.38% accuracy. This work presents a charge‐retained approach for continuous sensing with triboelectric‐based sensors, offering valuable insights for developing future multifunctional HMI and sign language recognition systems. The proposed smart glove system, with its dual‐mode sensing and AI integration, holds great potential for revolutionizing various domains and enhancing user experiences.

## Graphical abstract

A smart glove system named Triboelectric‐Inertial Dual‐Mode Sensing Glove (TI‐Glove) is proposed to enhance human‐machine interaction. It features a charge‐retained strategy that mitigates signal attenuation in triboelectric sensors, enabling accurate continuous finger movement measurement. The integration of inertial data and artificial intelligence techniques supports applications in robotics, virtual reality, and sign language recognition, achieving multifunctional and user‐friendly experiences.

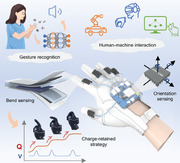

## 1 Introduction

Human‐machine interaction (HMI) is a rapidly evolving field, driven by advancements in artificial intelligence (AI), Internet of Things (IoT), and virtual reality (VR). As technology progresses, traditional interfaces such as keyboards and touchpads are proving to be inadequate for immersive interactions. To address this challenge, a novel solution is leveraging the dexterity of human hands, which opens up possibilities for applications such as complex robotic control, surgical robotics, and enhanced VR experiences.^[^ ^1^, ^2^, ^3^, ^4^ ^]^ Additionally, precise hand tracking can greatly benefit sign language, serving as a bridge to overcome communication barriers for individuals with speech impairments. Currently, hand motion tracking technologies can be categorized into two groups: one relies on high‐data‐rate and rigid schemes based on visual^[^ ^5^, ^6^, ^7^, ^8^ ^]^ and inertial measurement units (IMU),^[^ ^9^, ^10^ ^]^ the other utilizes flexible sensor schemes,^[^ ^11^, ^12^, ^13^ ^]^ such as resistive,^[^ ^14^, ^15^ ^]^ capacitive,^[^ ^16^, ^17^ ^]^ and fiber‐optic sensors.^[^ ^18^, ^19^ ^]^ While these systems successfully translate hand movements into digital signals for computer manipulation, they each come with certain limitations. Visual methods often struggle with occlusion and lighting issues, which can affect the accuracy and reliability of hand tracking. Traditional IMU gloves require multiple IMUs and demand high data processing capabilities, making them less practical for everyday use. Flexible sensors, such as resistive and capacitive types, have some advantages but require external excitation, which consumes some power.^[^ ^20^, ^21^ ^]^ Moreover, the manufacturing process for flexible sensors can be complex.^[^ ^16^, ^18^ ^]^

Triboelectric‐based sensors, renowned for their low cost, simple fabrication process, a wide range of material options, and zero‐power sensing, have been widely employed in the fields of wearable devices,^[^ ^22^, ^23^, ^24^, ^25^, ^26^, ^27^, ^28^, ^29^, ^30^, ^31^ ^]^ especially to capture finger bending motions with high sensitivity. However, since triboelectric‐based sensors are characterized by high internal impedance, and the output charge is highly susceptible to dissipation in the measurement circuit, there is still a great challenge to accurately measure the effective signal of the sensor. While high‐impedance electrometers have been widely used to precisely measure triboelectric sensor output voltage,^[^ ^24^, ^30^ ^]^ this approach is costly and impractical for portable devices. To achieve portable measurements, researchers have turned to commercial analog‐to‐digital converters (ADCs)^[^ ^32^, ^33^ ^]^ or custom printed circuit boards (PCBs),^[^ ^27^, ^34^ ^]^ but these devices' low internal impedance often leads to charge leakage in the measurement circuitry, causing acquired triboelectric signals to only capture transient action signals. Such information‐deficient sensing signals cannot accurately reflect the continuous human body movement, limiting potential applications of triboelectric‐based sensors. In an attempt to address this limitation, Lu et al. proposed using high‐resistance at GΩ level for precise voltage signal measurement,^[^ ^35^ ^]^ but this approach requires a high resistance resistor. Sun et al. suggested integrating the voltage to obtain continuous signals.^[^ ^28^ ^]^ Nonetheless, this method still fails to fully resolve the dissipative loss of triboelectric charges within the measuring circuit. Therefore, there is still an urgent need to develop a portable measurement scheme that can retain the output charge of triboelectric‐based sensors and acquire continuous sensing signals.

In this research, a smart glove system named Triboelectric‐Inertial Dual‐Mode Sensing Glove (TI‐Glove) is proposed. The TI‐Glove incorporates five triboelectric‐based sensors for finger bending motion detecting, an IMU for hand orientation capturing, a novel signal processing circuit based on charge measurement method for retaining sensing charge, and a main control circuit with Wi‐Fi module for data acquisition and wireless communication. This highly integrated solution, which is capable of real‐time, precise, and continuous motion capture of hand movements with zero‐power bending sensing, provides the advantages of portability and multifunctionality compared to similar systems (see Table S1, Supporting Information). In the proposed glove system, the charge‐retained signal processing circuit could effectively address the charge leakage issue of triboelectric‐based sensors, enabling continuous detection of finger movements. By combining triboelectric and inertial sensing, the system achieves a well‐balanced mix of versatility and system simplicity. This advancement has unlocked a wide range of applications, such as complex robot control, VR interaction, smart home lighting adjustments, and intuitive interface operations, showcasing the device's potential for low‐cost, multifunctional human‐machine interaction scenarios. Furthermore, an AI‐integrated sign language recognition system is developed for individuals with speech impairments. Through a fusion algorithm incorporating dual‐mode sensor information, an impressive recognition accuracy of 99.38% on a mixed dataset has been achieved, successfully recognizing complex gestures involving both finger bending and hand orientation. This achievement provides new insights into the processing of triboelectric sensing signals and underscores the potential applications of future smart gloves in human‐machine interaction and sign language recognition contexts.

## 2 Results and Discussion

### 2.1 Structure and Design of the TI‐Glove

**Figure 1:**
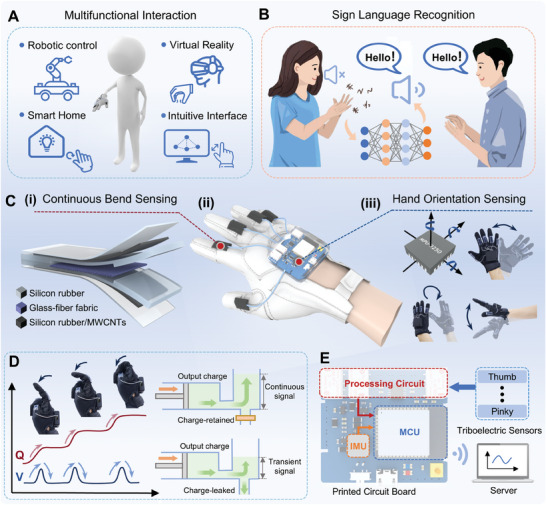
The intelligent system of the TI‐Glove with triboelectric‐inertial dual‐mode sensing. A) Schematics of multifunctional human‐machine interaction for robotic control, virtual reality, smart home, and intuitive interface. B) Schematics of sign language recognition system. C) (i) The detailed structure of the flexible triboelectric‐based sensor. (ii) Overall structure of the TI‐glove. (iii) Hand orientation sensing achieved by IMU. D) Comparison of signals in charge acquisition and voltage acquisition methods for triboelectric‐based sensors during continuous bending, and the charge‐retained circuit prevents leakage of the output charge from triboelectric‐based sensor, enabling continuous signal capture. E) Schematic block diagram of signal flow in the system.

This study introduces a smart glove system integrating triboelectric‐based sensors and IMU, which is designed to support diverse HMI applications, including robot control, VR interaction, smart home control, and intuitive interface operations (**Figure** **1**A). In addition, the system is capable of efficiently recognizing sign language information represented by complex gestures (Figure 1B). The core components of the system include five flexible triboelectric‐based sensors and an enhanced PCB with built‐in IMU and related functional circuits, which not only enables continuous finger bending sensing and hand gesture sensing but also signal processing and data transmission without relying on external wires or additional measurement devices (Figure 1C). The physical diagrams of the overall system can also be found in Figure S3 (Supporting Information).

The triboelectric‐based sensor features a fully flexible design. Silicone rubber serves as the substrate for the sensor, forming a cavity within it, as shown in Figure 1C(i). Glass‐fiber fabric is attached to one side of the cavity to form a silicone rubber and glass‐fiber fabric triboelectric pair. Additionally, the flexible electrodes using silicone rubber with multi‐walled carbon nanotubes (MWCNTs) were fabricated,^[^ ^36^ ^]^ as detailed in Figure S4 (Supporting Information). To safeguard the performance of the sensor from external environment, additional layer of silicone rubber was used to encapsulate the sensor, with the fabrication process shown in Figure S5 (Supporting Information). The sensor exhibits good mechanical performance, maintaining its flexibility during stretching, bending, and twisting tests (Figure S6, Supporting Information), thereby enabling a good shape fit with the fingers during use.

The enhanced PCB integrates an IMU, signal processing circuit, analog‐to‐digital converter circuit, and main control circuit, achieving signal processing, analog‐to‐digital conversion, wireless transmission, and independent power supply functionalities. Figure 1D compares the signals obtained using the charge acquisition method versus the conventional voltage acquisition approach for triboelectric‐based sensors during continuous bending. Conventional voltage acquisition approach is plagued by charge leakage, resulting in the capture of only transient signals and rapid charge dissipation. In contrast, the signal processing circuit based on charge‐retained circuit effectively prevents charge leakage from triboelectric‐based sensors during continuous bending, enabling continuous signal capture. With a compact and lightweight design, the entire circuit weighs only 18.2 g (including battery), making it ideal for portable applications.

The signal flow within this system is illustrated in Figure 1E. Five triboelectric‐based sensors capture the finger bending motion signals, which are then routed through the signal processing circuit to the microcontroller unit (MCU). Simultaneously, the IMU detects changes in the hand orientation, with its output data directly transmitted to the MCU. The processed sensor signals are wirelessly transmitted to the server host. Subsequently, leveraging the signal characteristics, multifunctional human‐machine interaction control of the corresponding terminal can be achieved.

### 2.2 Working Mechanism and Characterization of Triboelectric‐Based Sensors

**Figure 2:**
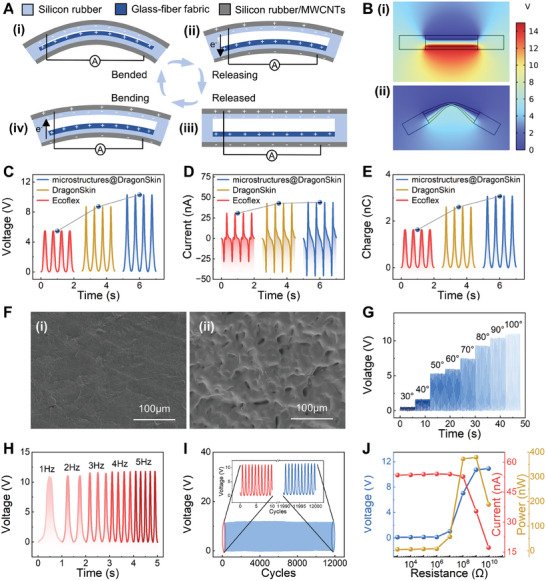
Working mechanism and characterization of triboelectric‐based sensor. A) Schematic illustration of the working mechanism of the triboelectric‐based sensor. B) Electric potential distribution simulation of the triboelectric‐based sensor when (i) released and (ii)bended. C–E) Open‐circuit voltage, short‐circuit current, and transferred charge of the triboelectric‐based sensor made by Ecoflex, DragonSkin, and DragonSkin with microstructures. F) SEM image of (i) flat surface and (ii) surface with microstructures. G) Open‐circuit voltage at different bending angles. (H) Frequency dependence of the open‐circuit voltage. I) Mechanical durability test for up to 12000 continuous bending cycles. J) Dependence of the voltage, current, and power output on the external load resistance.

The operational principle of the triboelectric‐based sensor within this system, employing the dual‐electrode contact‐separation mode, is illustrated in **Figure** **2**A. And glass‐fiber fabric with a low electron affinity is used as a positive triboelectric material.^[^ ^37^ ^]^ When the sensor undergoes bending, the contact area between the silicone rubber (serving as negative triboelectric material) and the glass‐fiber fabric expands. This movement triggers the triboelectric effect, facilitating the transfer of charge from the surface of the glass‐fiber fabric to the surface of the silicone rubber. Consequently, there is a potential alteration at the output electrode, inducing electron flow and thereby generating an electric signal output through electrostatic induction. To visualize the potential distribution changes of the sensor during this process, electrical simulations were conducted using COMSOL Multiphysics software, as shown in Figure 2B, which illustrates the sensor's potential distributions during release (i) and bending (ii).

To optimize signal output performance of the sensor, two different silicone rubber materials, Ecoflex and DragonSkin, were selected for sensor fabrication and comparative analysis. Experimental data revealed that DragonSkin exhibited higher open‐circuit voltage, short‐circuit current, and transferred charge compared to Ecoflex. Additionally, microstructures were created on the surface of the DragonSkin triboelectric layer using 1000‐grit sandpaper,^[^ ^38^, ^39^ ^]^ which effectively enhanced the sensor's output performance by increasing the contact area,^[^ ^40^ ^]^ as depicted in Figure 2C–E. Scanning electron microscopy (SEM) images in Figure 2F showcase the surface of the DragonSkin friction layer without microstructures (i) and with microstructures (ii).

In order to evaluate the triboelectric‐based sensor's capability in detecting finger bending, a linear motor was employed as testing platform to assess the sensor's electrical properties (Figure S7, Supporting Information). Figure 2G illustrates variation in open‐circuit voltage of the sensor across bending angles ranging from 30° to 100°. Notably, as the bending angle increases, the output voltage signal correspondingly escalates, demonstrating the sensor's capability to discern various finger bending states effectively. Figure S8 (Supporting Information) demonstrates the repeatability and consistency of the sensor output over multiple measurements. Figure 2H displays the variation in open‐circuit voltage at different bending frequencies. As shown in Figure S9 (Supporting Information), the sensor exhibits a response time of ≈46 ms, enabling precise responses to high‐frequency bending motions of the finger. Furthermore, Figure 2I presents the sensor's stable open‐circuit voltage over 12000 cycles of continuous operation, indicating its robust mechanical durability, while Figures S10 and S11 (Supporting Information) present the long‐term stability and hydrolysis resistance of the sensor, respectively. The voltage, current, and power curves under various external load resistances are depicted in Figure 2J, with the sensor achieving a maximum output power of 379.4 nW at an external resistance of 1GΩ, indicating an internal impedance of ≈1GΩ.

### 2.3 Charge‐Retained Circuit for Acquiring Triboelectric Continuous Signal

**Figure 3:**
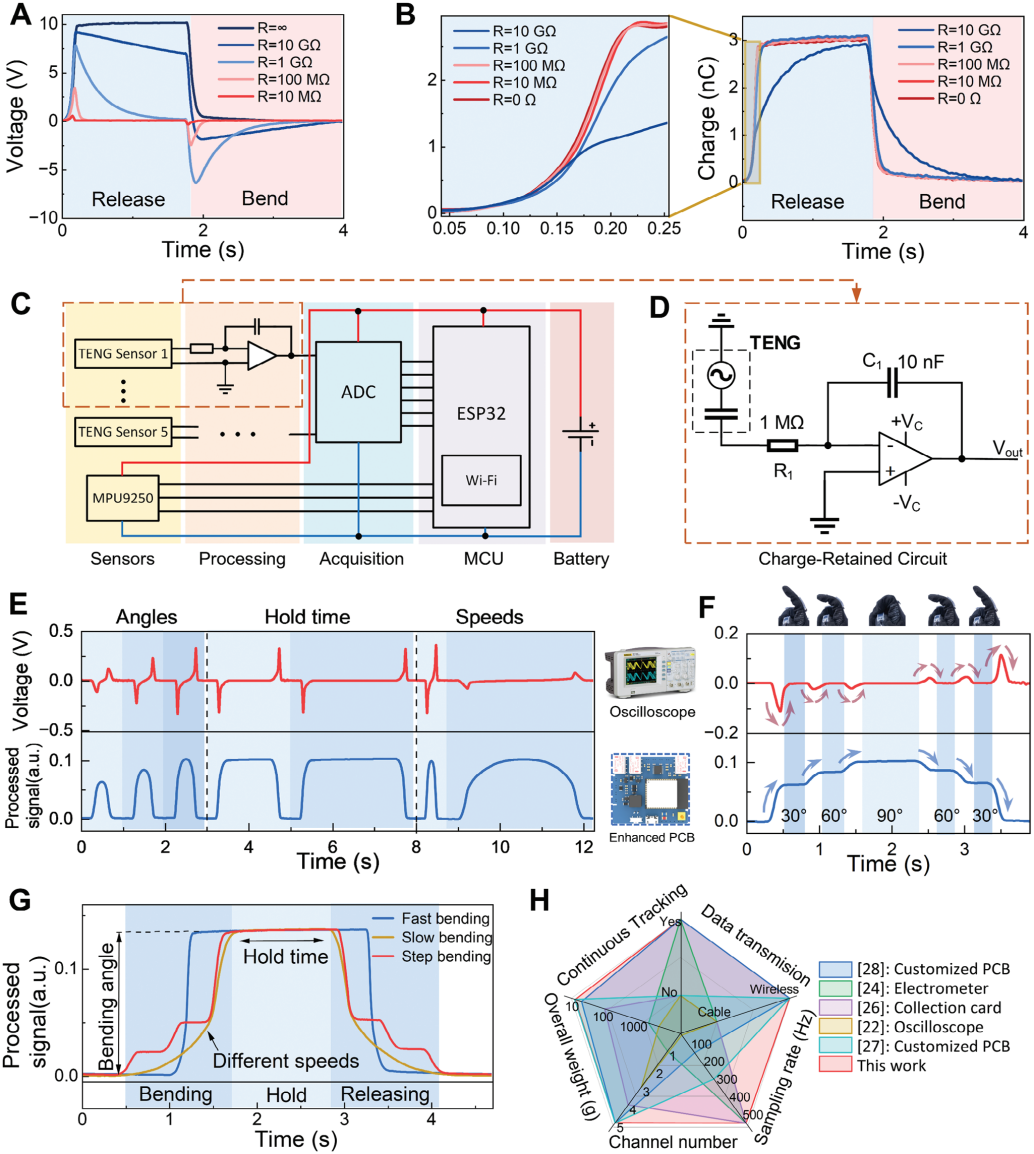
Analysis and portable acquisition of triboelectric sensing signals. A,B) Output voltage and charge waveforms of triboelectric‐based sensors under different external loads within one cycle. Inset: Enlarged view of the charge waveforms during the rising phase. C) Circuit diagram of the TI‐Glove system. D) Triboelectric signal processing circuit based on a charge measurement strategy. E) Comparison of triboelectric signals acquired through two strategies at different angles, hold times, and speeds during sensor bending. Top: Output voltage measured by an oscilloscope. Bottom: Signal acquired by the enhanced PCB. F) Comparison of signals acquired during step bending and releasing. G) Multi‐dimensional features of the bending motion extracted from the output signals, including bending angle, hold time, and bending speed. H) Comparison of various triboelectric signal measurement schemes.

Triboelectric‐based sensors have been extensively studied in existing literature, with a predominant focus on utilizing output voltage as the sensing signal.^[^ ^22^, ^23^, ^24^, ^25^, ^26^, ^27^, ^28^, ^32^ ^]^ To enable portable measurement of these signals, researchers commonly employ commercial ADCs^[^ ^32^, ^33^ ^]^ or customized PCBs^[^ ^27^, ^34^ ^]^ for triboelectric signals capturing. However, the high internal impedance of triboelectric‐based sensors, typically ≈1 GΩ, poses a significant challenge as it greatly influences the output signal of the measurement circuit. To study this challenge, different load resistors were connected to the output terminal of the sensor and the voltage waveform variations across the resistors were analyzed to reflect the influence of the internal impedance of the non‐ideal voltage measurement circuit on the sensor's output signal. Detailed experimental procedures are provided in Note S1 (Supporting Information). This impedance matching issue is revealed in **Figure** **3**A, where a decrease in external load on the sensor leads to a proportional reduction in output voltage, with an accelerated rate of attenuation. Consequently, when low‐impedance measuring instruments are employed, the triboelectric signal rapidly attenuates within the circuit, resulting in a pulse‐like waveform. This rapid attenuation has consequently restricted many previous studies to focusing on the analysis of intermittent signals, thereby limiting their ability to capture continuous signals.

In contrast to the output voltage, which is the primary focus of the aforementioned studies, the output charge signal from triboelectric‐based sensors remains relatively consistent under different external loads, making it a more suitable signal type for portable measurements. Thus, the output charge signal of the sensor under various external loads were measured, with experiment details provided in Note S2 (Supporting Information). As illustrated in Figure 3B, the waveform of the triboelectric‐based sensor output charge exhibits a notable slowdown in the rising rate only when subjected to an external load of 10 GΩ. The enlarged figure highlights the differences in the rising phase of the output charge waveform. Although an increase in external load resistance results in a slower rising speed of the waveform, this effect remains negligible when the external load is below 100 MΩ. This shows that as long as the internal impedance of the measurement circuit is low (e.g., less than 100 MΩ, which is easy to achieve in circuit design), the output charge signal of the triboelectric‐based sensor can remain stable and consistent in the circuit.

Our study introduces a lightweight triboelectric signal processing and transmission circuit based on charge measurement (Figure 3C). The analog signals are processed and converted to digital signals using an ADC before being transmitted to the MCU, an ESP32 module facilitating wireless signal transmission via Wi‐Fi. Additionally, an IMU is incorporated into the circuit, with its output signals directly sent to the MCU. The entire circuit is powered by a Li‐ion battery, ensuring complete portability in signal acquisition, processing, and transmission.

The schematic diagram of the triboelectric signal processing circuit is detailed in Figure 3D, integrating a precision operational amplifier (op‐amp) and a feedback capacitor to directly accumulate the charge generated by the triboelectric‐based sensor. When the sensor undergoes mechanical deformation, the generated charge creates a voltage across itself, which in turn drives a small current through resistor *R*_1_ toward the inverting input of the op‐amp. The op‐amp's high input impedance ensures minimal current flow into the inverting input, enabling effective charge accumulation on the integrating capacitor *C*_1_, which subsequently generates a continuous output voltage. If *Z_f_* represents the feedback loop impedance and *Z_s_* denotes the internal impedance of the sensor, the output voltage of the circuit can be expressed as:

(1) $$V_{\mathrm{out}}=-\frac{Z_{f}}{Z_{s}+R_{1}}V_{s}=-\frac{Z_{f}}{Z_{s}+R_{1}}\frac{Q_{s}}{C_{s}}$$

where *Q_s_* is the output charge of the triboelectric sensor, *V_s_* represents the ideal output voltage of the sensor (open‐circuit voltage), and *C_s_* is the internal capacitance of the sensor. Given that *Z_s_* is ≈1GΩ, which is significantly larger than *R*_1_, we have:

(2) $$V_{\mathrm{out}}=-\frac{Z_{f}}{Z_{s}}\frac{Q_{s}}{C_{s}}=-\frac{Q_{s}}{C_{1}}$$

Therefore, the output voltage of the processing circuit is directly proportional to the output charge of the triboelectric‐based sensor and inversely proportional to the capacitance in the feedback loop.

To demonstrate the role of the charge‐retained circuit in capturing continuous signals of triboelectric‐based sensor, a linear motor was employed as the testing platform. Different sensor bending angles, holding time, and bending speeds were configured for the tests, with the output signals of the triboelectric sensor being compared using an oscilloscope, with an internal impedance of 100 MΩ, and the enhanced PCB integrated the charge‐retained circuit as acquisition devices. As shown in Figure 3E, the signals captured by the oscilloscope exhibited a rapid decrease, forming an alternating pulse‐like signal, while the signal captured by the PCB not only distinguish between different bending angles, but also stably maintain the sensed signals when the sensor angle remains unchanged. Notably, during the slow bending and releasing processes of the sensor, the signal captured by the oscilloscope exhibited only minor peaks at the beginning and end stages, whereas the PCB can retain the output charge of the sensor throughout the entire process, thus capturing a continuous signal, which demonstrates the superiority of this approach in accurately acquiring triboelectric signal. Figure S12 (Supporting Information) compares the triboelectric sensor output charge signal with the enhanced PCB output signal during the same test process, showing highly consistent under different test conditions, aligning with theoretical analysis.

To further illustrate the ability of this processing circuit to capture continuous motions, Figure 3F presents a signal comparison between the two measurement methods when the sensor undergoes stepwise bending and releasing. The sensor was controlled to bend at a small angle and then held for a period, with different bending states shown in Figure S13 (Supporting Information). The oscilloscope measurement signal decay rapidly after each individual bending or releasing step, reflecting only the degree of bending at each step. In contrast, the enhanced PCB with charge‐retained circuit was able to avoid charge leakage and thus capture continuous angle changes throughout the entire process.

Drawing from the above experimental results, the multi‐dimensional characteristics of the sensor output signal can be extracted using this processing circuit for more comprehensive bending sensing. As shown in Figure 3G, the amplitude of the output signal can characterize the bending angle of the finger, while the duration of the output signal maintenance corresponds to the duration the finger remains bent. Additionally, the speed of finger bending and releasing can be determined by comparing the slopes of the rising and falling curves of the output signal.

The enhanced PCB, including the battery, boasts a remarkably low weight of only 18.2 g (Figure S14, Supporting Information), with a maximum sampling rate of 500 Hz. This configuration enables portable multi‐channel acquisition of continuous triboelectric signals. The physical diagrams of the enhanced PCB are shown in Figure S15 (Supporting Information), with a comparison of its related performances,^[^ ^22^, ^24^, ^26^, ^27^, ^28^ ^]^ including continuous signal capture capability, data transmission mode, sampling rate, number of channels, overall weight with other measurement methods presented in Figure 3H and Table S2 (Supporting Information). The estimated power consumption of each module and the overall system is provided in Table S3 (Supporting Information).

### 2.4 Application of TI‐Glove in Multifunctional Human‐Machine Interaction

To enhance the functionality of the smart glove, a single IMU chip is integrated into the PCB for reflecting changes in hand posture. By connecting five triboelectric‐based sensors to the enhanced PCB with integrated IMU, the TI‐Glove successfully realizes the acquisition, processing, and wireless transmission of triboelectric‐inertial dual‐mode sensing signals, based on which the real‐time continuous control of the robot is realized.

**Figure 4:**
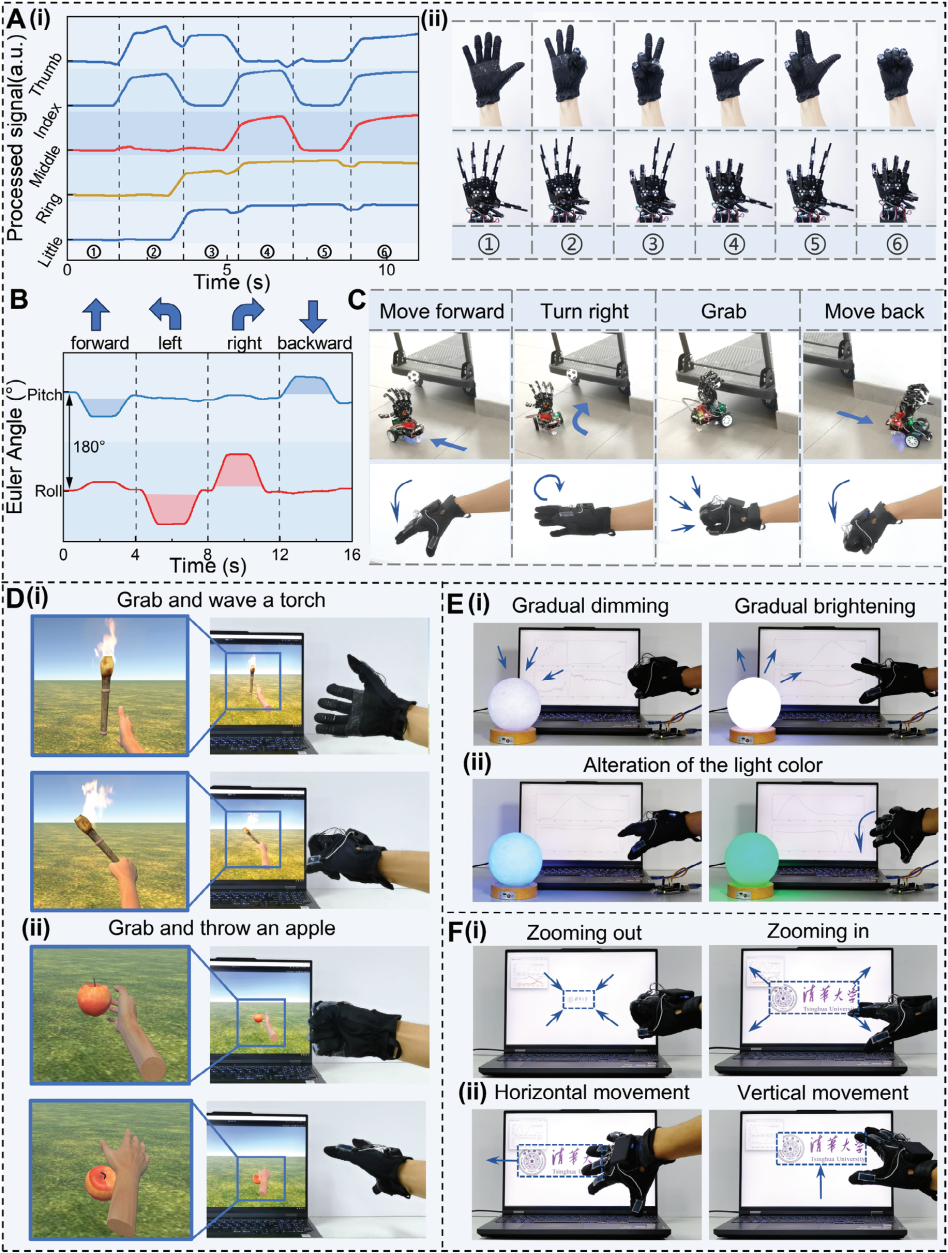
Demonstrations of TI‐Glove in multifunctional human‐machine interaction. A) Robotic hand control. (i) Real‐time signal output of the triboelectric‐based sensors for different gestures and (ii) Photographs corresponding to human gestures and robotic hand motions. B) IMU signals for controlling robot's forward, backward, left, and right turns. C) Screenshots of the integrated demonstration showing the advanced robotic control for movement and grab. D) TI‐Glove enabled VR interactions, including (i) grabbing and waving a torch, (ii) grabbing an apple and throwing it. E) Demonstration of (i) adjusting light brightness smoothly and (ii) altering light color. F) Intuitive interface through hand movements, including control of image (i) zoom in/out and (ii) horizontal/vertical movement.

**Figure** **4**A and Movie S1 (Supporting Information) demonstrate the TI‐Glove's ability to capture gestures and control a robotic hand. When wearing TI‐Glove and executing various gestures, the five sensors autonomously output their respective sensing signals, representing the bending degree of each finger, as shown in Figure 4A(i). This signal can be used to control the corresponding fingers of the robotic hand, to achieve various degrees of bending (Figure S16, Supporting Information), enabling complex robot gesture operations. The response of corresponding robotic hand actions to different gestures is shown in Figure 4A(ii). Figure S17 (Supporting Information) presents the output signals of the triboelectric‐based sensors under three distinct bending angles, two different holding times, and two diverse bending speeds, showing the multi‐dimensional characteristics reflected in the signals. Precise synchronization between robotic hand and human hand movements can be achieved based on the information provided in the signals (Movie S2, Supporting Information).

To illustrate the integrated application of TI‐Glove in advanced robot control, the output signals from the triboelectric‐based sensors were utilized to manipulate the gripping of the robotic hand, while the IMU signals controlled the direction of robot movement (Figure 4B). Figure 4C and Movie S3 (Supporting Information) showcase the potential utility of TI‐Glove in remote synchronous control of robot for hazardous material handling. Personnel can remotely guide the robot to a specified location through hand gestures, synchronize finger movements to accurately grasp the target object, and relocate the object to a safe area.

By combining triboelectric signals and IMU data, application scope of TI‐Glove has been expanded in the field of human‐machine interaction, highlighting its versatile capabilities. By utilizing the Unity platform, human hand gestures were simulated in a virtual environment. Here, the 5‐channel continuous triboelectric signals aligned the virtual hand movements with the bending of human finger (Figure S18A, Supporting Information), while the IMU data captured the real‐time orientation of the human hand (Figure S18B, Supporting Information). As exhibits in Figure 4D and Movie S4 (Supporting Information), TI‐Glove enabled tasks such as grasping and waving a torch in the virtual world (Figure 4D(i)), along with picking up an apple and tossing it away (Figure 4D(ii)). Figure 4E and Movie S5 (Supporting Information) demonstrate the potential of TI‐Glove in the domain of smart home applications. Varied angles of finger bending regulated the brightness of lights (Figure 4E(i)), whereas tilting the hand altered the light colors (Figure 4E(ii)). Notably, owing to TI‐Glove's ability to capture continuous triboelectric signals, light adjustment can allow for gradual changes in brightness beyond binary “on” and “off” states, enabling a smooth transition in light intensity through finger bending. Figure 4F and Movie S6 (Supporting Information) showcase the promising use of TI‐Glove as an intuitive interaction interface. Tightening the fingers zoomed out the image, while loosening them zoomed in (Figure 4F(i)). Similarly, adjustments in the overall hand orientation resulted in horizontal or vertical movements of the image accordingly (Figure 4F(ii)). These diverse application scenarios underscore the extensive potential of TI‐Glove in advanced robot control, VR interactions, smart homes, and intuitive interfaces.

### 2.5 AI‐Enabled Sign Language Recognition System

In order to achieve barrier‐free communication between speech impaired and able‐bodied people, there is a need to develop an effective sign language translation system. Numerous studies have indicated that triboelectric‐based sensors can be utilized to capture hand gestures, thereby achieving high‐precision sign language translation.^[^ ^27^, ^38^, ^41^ ^]^ While these sensors have shown success in recognizing a variety of gestures, the nuances of sign language extend beyond just hand movements to include hand orientations. To address the variations in sign language that stem from differences in hand orientations, an integrated approach is required. By combining triboelectric bending sensors with inertial sensors, a more comprehensive system can be developed to recognize complex sign language expressions involving finger bending and hand orientations.

**Figure 5:**
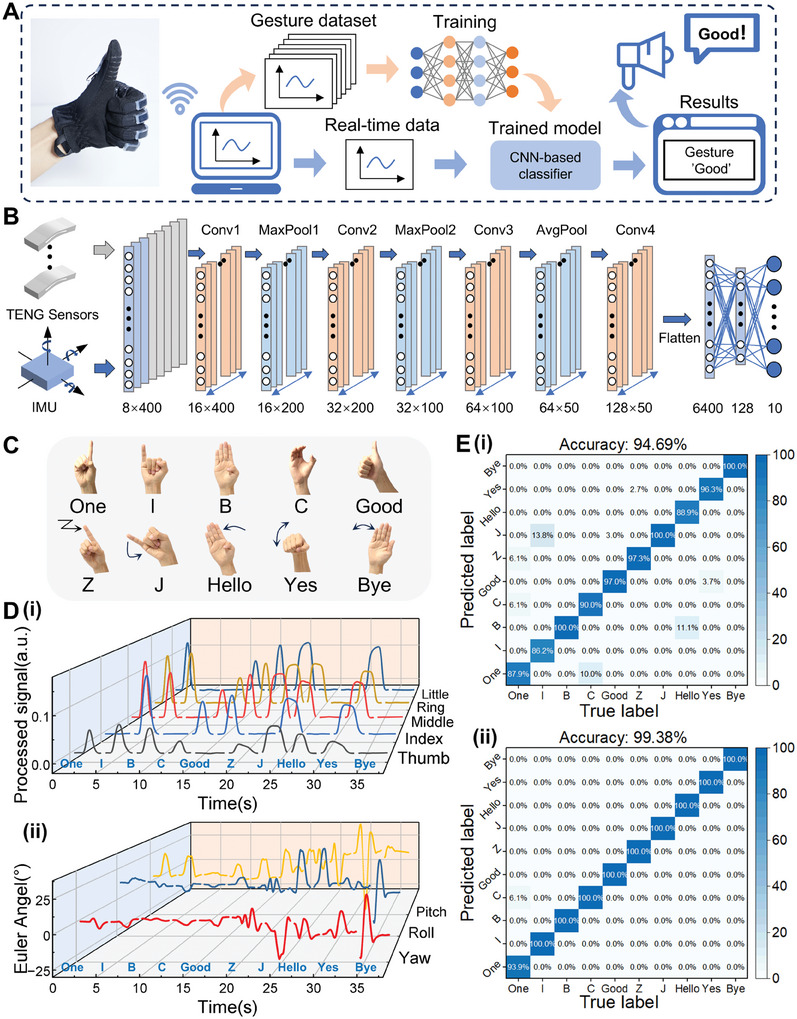
AI‐enabled sign language recognition system. A) Process flow of the training and real‐time sign language gestures identification. B) Schematic diagram of the overall structure of CNN. C) Illustration of selected 10 American sign language gestures. Above: Gestures containing basic finger bending. Below: Gestures containing finger bending and hand orientation. D) (i) Triboelectric‐based sensors and (ii) IMU output signals generated by 10 sign language gestures in the dataset. E) Confusion map of recognizing 10 sign language gestures (i) using only finger bending data and (ii) fusing finger bending data and hand orientation data.

Deep learning algorithms were employed to integrate dual‐mode sensing data from triboelectric‐based sensors and inertial sensor to achieve intelligent recognition of complex hand gestures involving finger bending and hand orientation. The flowchart depicting the training and real‐time recognition process is presented in **Figure** **5**A. Initially, sensing data from various gestures was collected using TI‐Glove during training phase at a sampling rate of 100 Hz. This data was then segmented, normalized, and used to form the dataset for training deep learning model. Detailed data collection and dataset configuration can be found in the experimental section. For the recognition phase, real‐time sensor data was input into the pre‐trained model, allowing the system to identify gesture types and produce speech output. In order to effectively handle the temporal sequence signals from multi‐channel sensors, a 1D convolutional neural network (1D‐CNN) model was utilized as the training model, as demonstrated by its overall architecture in Figure 5B, along with the detailed parameters in Table S4 (Supporting Information). The network took a combination of 5‐channel triboelectric‐based sensor signals and 3‐channel IMU‐captured pose angle data as inputs, and classification results were obtained after passing through 4 convolutional layers, 3 pooling layers, and 2 fully connected layers.

The gesture dataset comprised 10 American Sign Language gestures, as displayed in Figure 5C. These gestures were divided two categories: five involving only finger bending (upper half) and five incorporating both finger bending and hand orientation movements (lower half). Figure 5D illustrates the output signals of various gesture samples, with Figure 5D(i) representing signals from the 5‐channel triboelectric‐based sensors, and Figure 5D(ii) showcasing the 3‐axis attitude angle data captured by the IMU. A total of 1000 samples were recorded for the ten gesture categories, with the samples shuffled and split randomly into training and testing sets at a ratio of 7:3.

Figure 5E compares the recognition performance when solely using triboelectric‐based sensors versus integrating triboelectric‐inertial sensing for complex sign language recognition. The confusion map reveals a recognition accuracy of 94.69% when only using triboelectric‐based sensors to capture finger bending (Figure 5E(i)). Notably, considerable misclassifications were observed among gestures “I” and “J”, as well as “Hello” and “B”, indicating challenges in effectively classifying complex gestures with similar finger bending patterns but distinct hand orientations solely based on bending sensing data. Conversely, upon integrating finger bending and hand orientation data, a significant enhancement in gesture classification performance was achieved, with a stable accuracy rate of 99.38% after 400 epochs. The classification confusion matrix for the 10 categories is presented in Figure 5E(ii), while Figure S19 (Supporting Information) showcases the accuracy and loss curves of the model during training and testing. Through the fusion model of triboelectric and inertial sensors, the sign language recognition system successfully identified complex sign language involving both finger bending and hand orientation, as evidenced in Movie S7 (Supporting Information). The performance benchmarks of various machine learning architectures on this dataset are presented in Table S5 (Supporting Information).^[^ ^27^, ^42^, ^43^ ^]^

## 3 Conclusion

We have developed a TI‐Glove with triboelectric‐inertial dual‐mode sensing, to achieve a more intuitive and natural interactive experience, capturing both finger bending and hand orientation simultaneously. The functionalities are integrated into a single glove system, providing higher integration and a wider range of capabilities than current solutions. The output charge leakage problem of triboelectric sensors has been analyzed, and a lightweight signal processing circuit with charge‐retained capability has been proposed to realize portable measurement of continuous changes in triboelectric‐based sensor signals. The system utilizes the dual‐mode information from continuous triboelectric signals and inertial sensing signals to enable various human‐machine interaction functions, including advanced robot control, VR interaction, lighting control, and intuitive interface operation. Additionally, an AI‐enabled sign language recognition system has been developed for the speech impaired, leveraging the fusion of triboelectric and inertial sensing data to improve recognition accuracy of complex gestures involving finger bending and hand orientation. This system achieves a recognition rate of 99.38% for 10 gestures. The proposed TI‐Glove system is characterized by its simple design, low cost, and high level of integration, with charge‐retained circuit offering new insights into portable measurement methods for triboelectric‐based sensors. Application demonstrations showcase its potential across diverse fields such as industrial manufacturing, entertainment, smart home technologies, and disability assistance.

## 4 Experimental Section

### Fabrication of the Flexible Electrode

The fabricated flexible electrodes were composed of a mixture of silicone rubber and MWCNTs, possessing both good flexibility and stretchability, as well as excellent conductivity. The fabrication process was depicted in Figure S4 (Supporting Information). The A and B components of silicone rubber (DragonSkin 10 SLOW, Smooth‐on) were mixed in a 1:1 ratio, followed by the addition of MWCNTs (purity 99%, Shenzhen Suiheng Technology Co.), at a mass ratio of 1:100 relative to the silicone rubber. Dispersant (PVP, Suzhou Tanfeng Graphene Technology Co.) was then added at a mass ratio of 1:10 relative to the added MWCNTs. The mixture was stirred using an electric stirrer at a speed of 1200 rpm for 1 min. Subsequently, the mixture was scraped on a 3D‐printed mold and cured at room temperature for 4 h. The cured film was then peeled off from the mold for further use.

### Fabrication of the Triboelectric‐Based Sensor

The fabrication process of the triboelectric‐based sensor is shown in Figure S5 (Supporting Information). A 3D‐printed mold with grooves at both ends was prepared for fabricating the sensor substrate. The A and B components of silicone rubber (DragonSkin 10 SLOW, Smooth‐on) were mixed in a 1:1 ratio and poured into the mold. The mixture was then cured at room temperature for 4 h to form the sensor substrate. The pre‐fabricated flexible electrode was placed on the back of the substrate, and another layer of silicone rubber mixture was applied for encapsulation. After curing again, the fabrication of the sensor with a single‐sided structure was completed. Subsequently, glass‐fiber fabric was pasted on one side, while the other side remained untreated. The structures on both sides were then fixed together to complete the sensor fabrication.

### Glove Configuration

The prepared triboelectric‐based sensors were affixed to the joints of commercial gloves using silicone adhesive (Sil‐Poxy, Smooth‐on). Conductive wires were then used to connect the triboelectric‐based sensors to the input ports of enhanced PCB. Subsequently, the PCB was secured onto the back of the hand.

### Characterization and Measurement

In the performance characterization of the triboelectric‐based sensor, a linear motor (LinMot P01‐37×120‐C_C1100) was employed to provide stable and consistent motion. An electrometer (version Keithley 6514, impedance > 200 TΩ, The Keithley Inc.) was utilized for measuring the sensor's open‐circuit voltage, short‐circuit current, and transferred charge, while data acquisition was conducted using the NI 9215 acquisition card. For signal comparison experiments, an oscilloscope (MS0 2024B, Tektronix Inc.) and the enhanced PCB were employed for signal acquisition. In demonstration experiments, the enhanced PCB were used for signal acquisition, and Python 3.8 was used to build the software platform for real‐time signal reception, analysis, and processing.

### Methods for Advanced Robot Control

The enhanced PCB collected sensing signals, which were wirelessly received via Python for analysis. Subsequently, the control commands were transmitted to the robot through a wireless serial module (HC‐12, Guangzhou Huicheng Information Technology Co.), to control the robot to perform finger bending and moving tasks. The output signals from the five triboelectric‐based sensors were normalized to correspond to the bending angles of the robot's five fingers. The IMU signals were used to control the robot's motion, with specific rules dictating movement based on the hand's orientation. For instance, when the hand leans forward/backward (i.e., the IMU output pitch angle is less than 0/greater than 0), the robot moves forward/backward. Similarly, when the hand tilts to the left/right (i.e., the IMU output roll angle is less than 0/greater than 0), the robot shifts left/right, as shown in Figure 4B. Moreover, the robot halts its movement when the hand remains horizontal.

### VR Interaction System

The enhanced PCB in the TI‐glove captures the sensor signals, which are then transmitted wirelessly to the host via Wi‐Fi. A Python program on the host reads the transmitted data and performs signal normalization. The normalized data is sent to the Unity software through a socket connection. Within Unity, models of the hand were created and interactive objects, and a C# script was developed to translate the sensor signals into commands that control the model's movements.

### Lighting Control System

The TI‐glove captures sensor signals and wirelessly transmits them to the host. The signals are then converted into lighting control commands using a development board (Arduino Uno), which adjusts the brightness and color of the lights through an infrared module.

### Data Collection and Dataset Configuration

The enhanced PCB captured sensing signals generated by different gestures, including five triboelectric‐based sensors and an IMU 3‐axis attitude angle output. Each gesture was repeated 100 times, lasting 4 s each time, with a sampling frequency of 100 Hz. Using Python, a segmentation window of 400 data points was applied to extract signal fragments. Consequently, each dataset sample comprised 400 data points across 8 signal channels, resulting in a total of 1000 samples for 10 gestures. To equalize the distribution range of the triboelectric‐based sensor signals and IMU 3‐axis attitude angles in the samples, a max‐min normalization technique was utilized. This approach aimed to mitigate the adverse effects of varied magnitudes on the training effectiveness. Subsequently, the normalized samples underwent randomization to construct a dataset, with 70% used for training and 30% for testing.

### Sign Language Recognition System Workflow

Real‐time sensor signals captured during gesture movements are wirelessly transmitted to the server host via a Wi‐Fi module integrated into the main control circuit of the smart glove system. On the server, Python is used to receive sensor data, which is then segmented into 400‐point fragments from the continuous data streams generated by the sensors. The signal fragments are then normalized and input into the trained 1D‐CNN model for decoding. The model automatically extracts signal features and classifies the sensor data into one of ten predefined categories. Subsequently, the recognized gesture category was displayed on the screen, and the corresponding speech output was generated.

## Conflict of Interest

The authors declare no conflict of interest.

## Author Contributions

B.Y., J.C., and X.Q. contributed equally to this work. J.C. and B.Y. performed conceptualization; B.Y., J.C., X.Q., L.Y., Y.S., and J.S. performed methodology; B.Y. performed software; Y.S., J.C., and B.Y. performed hardware; B.Y., J.C., X.Q. and L.Y. performed investigation; B.Y., Z.B., and J.S. performed visualization; J.C. and X.Q. acquired funding acquisition; J.C. performed project administration; J.C. and L.J. performed supervision; B.Y. wrote the original draft; B.Y., J.C., and X.Q. wrote review and performed editing.

## Supporting information



Supporting Information

Supplemental Movie 1

Supplemental Movie 2

Supplemental Movie 3

Supplemental Movie 4

Supplemental Movie 5

Supplemental Movie 6

Supplemental Movie 7

## Data Availability

The data that support the findings of this study are available in the supplementary material of this article.
