# Supplementary material for: Triboelectric‐Inertial Sensing Glove Enhanced by Charge‐Retained Strategy for Human‐Machine Interaction
Source: Adv Sci (Weinh). 2024 Nov 22;12(3):2408689. doi: 10.1002/advs.202408689 (PMC11744583; doi:10.1002/advs.202408689)
Supplement: Supplementary file 1 — Supporting Information [file ADVS-12-2408689-s002.docx]

Supporting Information for

**Triboelectric-Inertial Sensing Glove Enhanced by Charge-Retained Strategy for Human-Machine Interaction**

*Bo Yang, Jia Cheng,* Xuecheng Qu, Yuning Song, Lifa Yang, Junyao Shen, Ziqian Bai, Linhong Ji*

*Corresponding author. Email: chengjia@tsinghua.edu.cn

**This PDF file includes:**

Notes S1 and S2

Figures S1 to S19

Tables S1 to S5

Legends for movies S1 to S7

**Other Supporting Information for this manuscript include the following:**

Movies S1 to S7

Note S1 The method for studying the effect of measuring circuit impedance on voltage output signal

Any non-ideal voltage measurement circuit possesses internal impedance, which can be theoretically simplified as an ideal voltmeter in parallel with a resistor, where the resistance value represents the equivalent internal impedance of the measurement circuit. To demonstrate the impact of a non-ideal voltage measurement circuit on the sensor output signal, an experimental circuit as depicted in Figure S1 was constructed. The experiment involved adjusting different external loads *R* on the sensor to simulate various internal impedances of the measurement circuit. A linear motor was utilized to provide consistent and stable bending motion to the sensor. The voltage signal output from the sensor was measured using a Keithley 6514 electrometer (with internal impedance >200 TΩ) set to the voltage range.


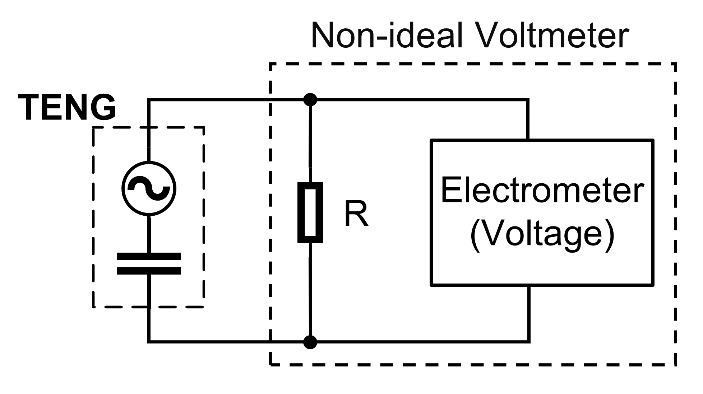


Figure S1. Non-ideal voltage measurement circuit measuring triboelectric sensor’s output.

Note S2 The method for studying the effect of measuring circuit impedance on charge output signal

The theoretical model of a non-ideal charge measurement circuit can be simplified as an ideal charge meter in series with a resistor, where the resistance value represents the equivalent internal impedance of the measurement circuit. To simulate the impact of a non-ideal charge measurement circuit on the sensor output signal, an experimental circuit as depicted in Figure S2 was constructed. In this study, a Keithley 6514 electrometer was used to measure the charge signal output from the sensor.


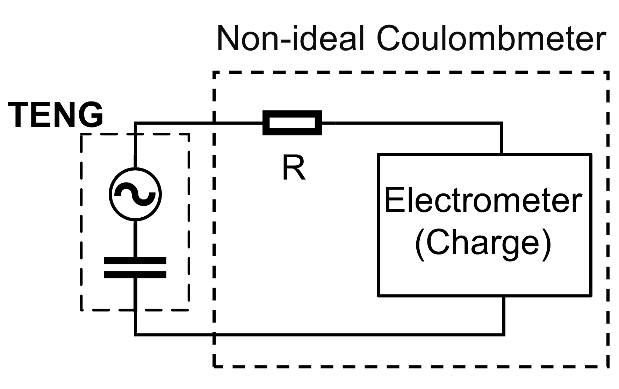


Figure S2. Non-ideal charge measurement circuit measuring triboelectric sensor’s output.


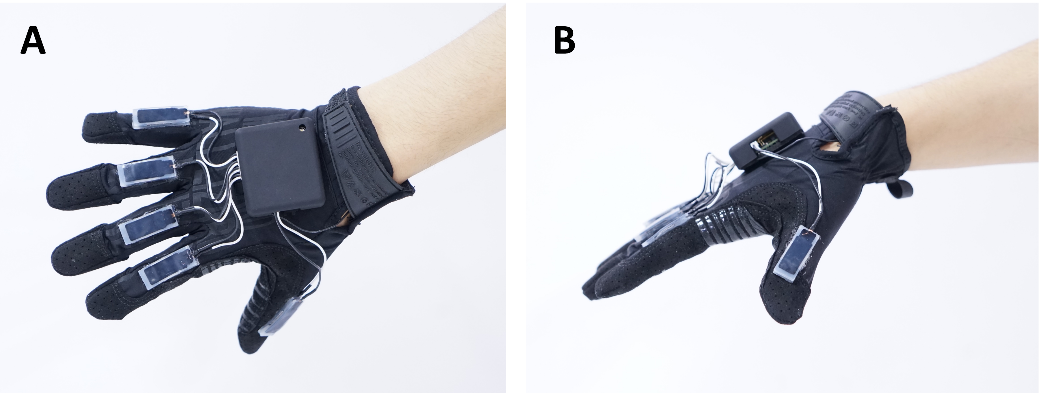


Figure S3. The physical diagrams of the overall system. (A) Top view. (B) Front view.


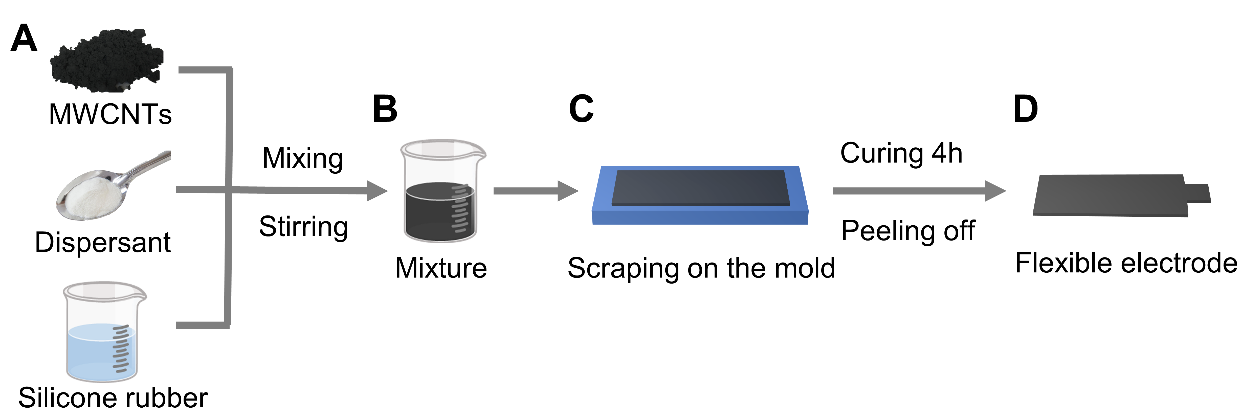


Figure S4. The detailed fabrication process of the flexible electrode. (A) Materials required for the preparation of flexible electrode. (B) Mixing and stirring to form a mixture. (C) Scraping on the mold and curing. (D) Peeling off to complete the flexible electrode fabrication.


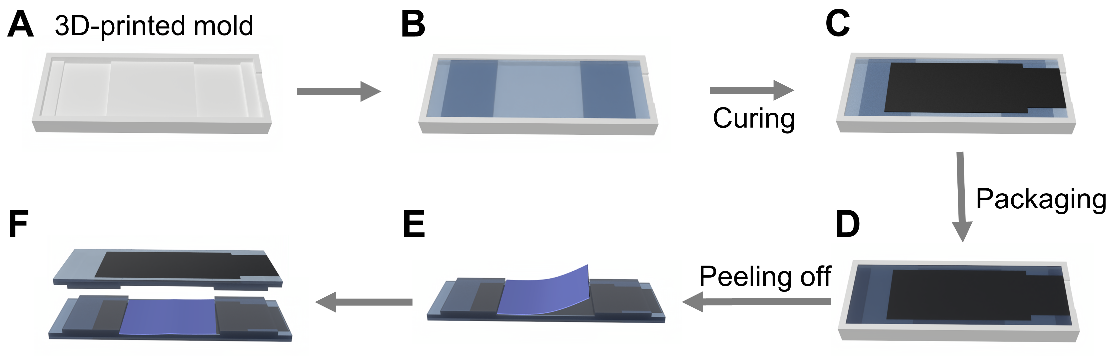


Figure S5. The detailed fabrication process of the triboelectric sensor. (A) A 3D-printed mold is prepared for fabricating the sensor substrate. (B) The silicone rubber mixture is poured into the mold. (C) The flexible electrode is placed on the back of the substrate. (D) Additional layer of silicone rubber mixture is applied for encapsulation. (E) The glass-fiber fabric is pasted on one side, while the other side remains untreated. (F) The structures on both sides are then fixed together.


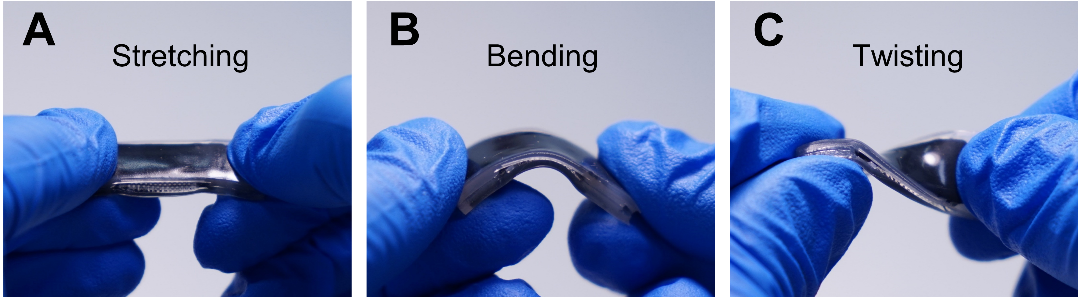


Figure S6. Photographs of the triboelectric sensor during mechanical testing: (A) stretching, (B) bending, and (C) twisting.

**
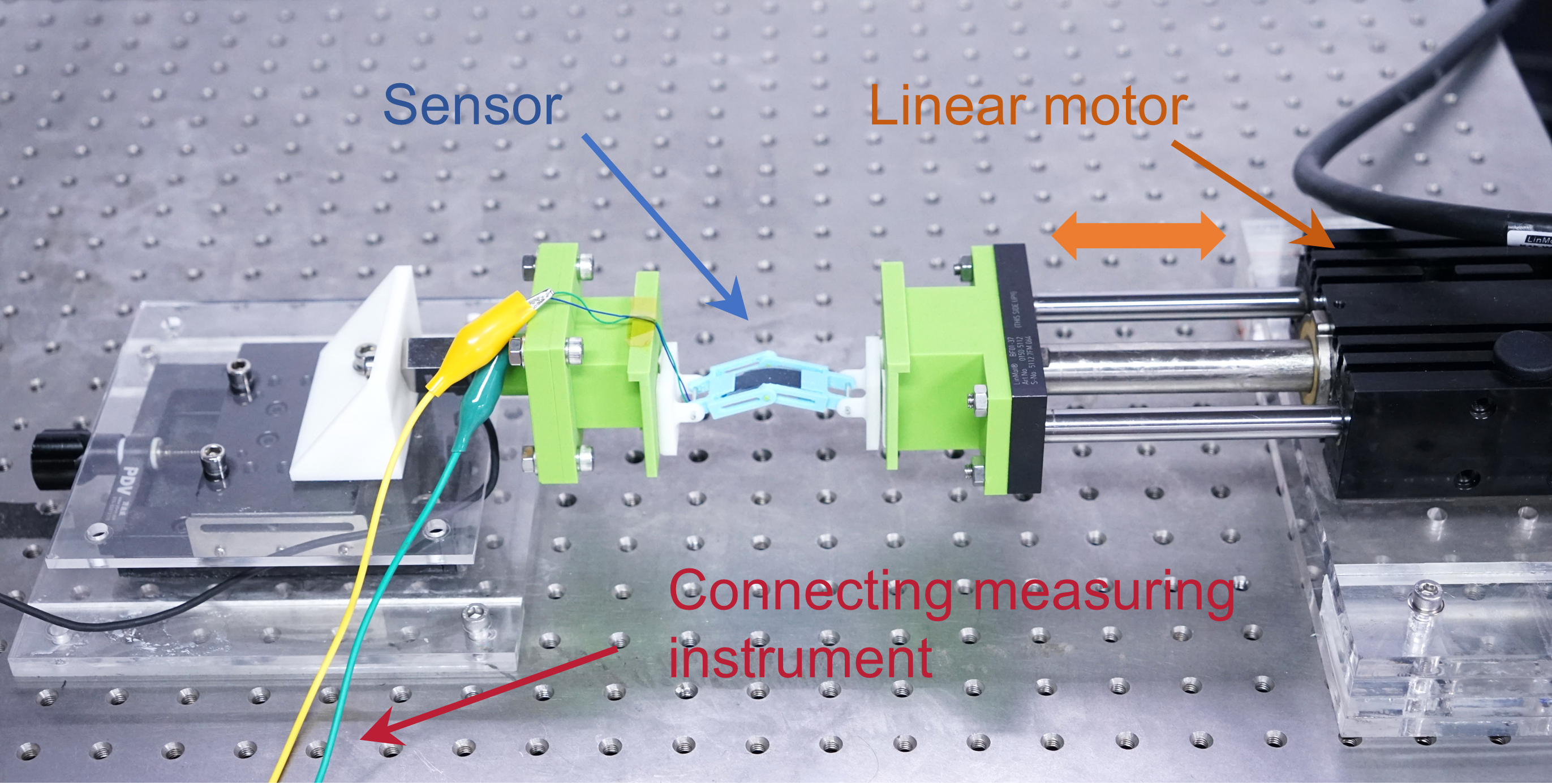
**

Figure S7. Photograph of the test platform, comprising includes a linear motor, a custom-designed sensor bracket and external measurement instruments connected via wires.


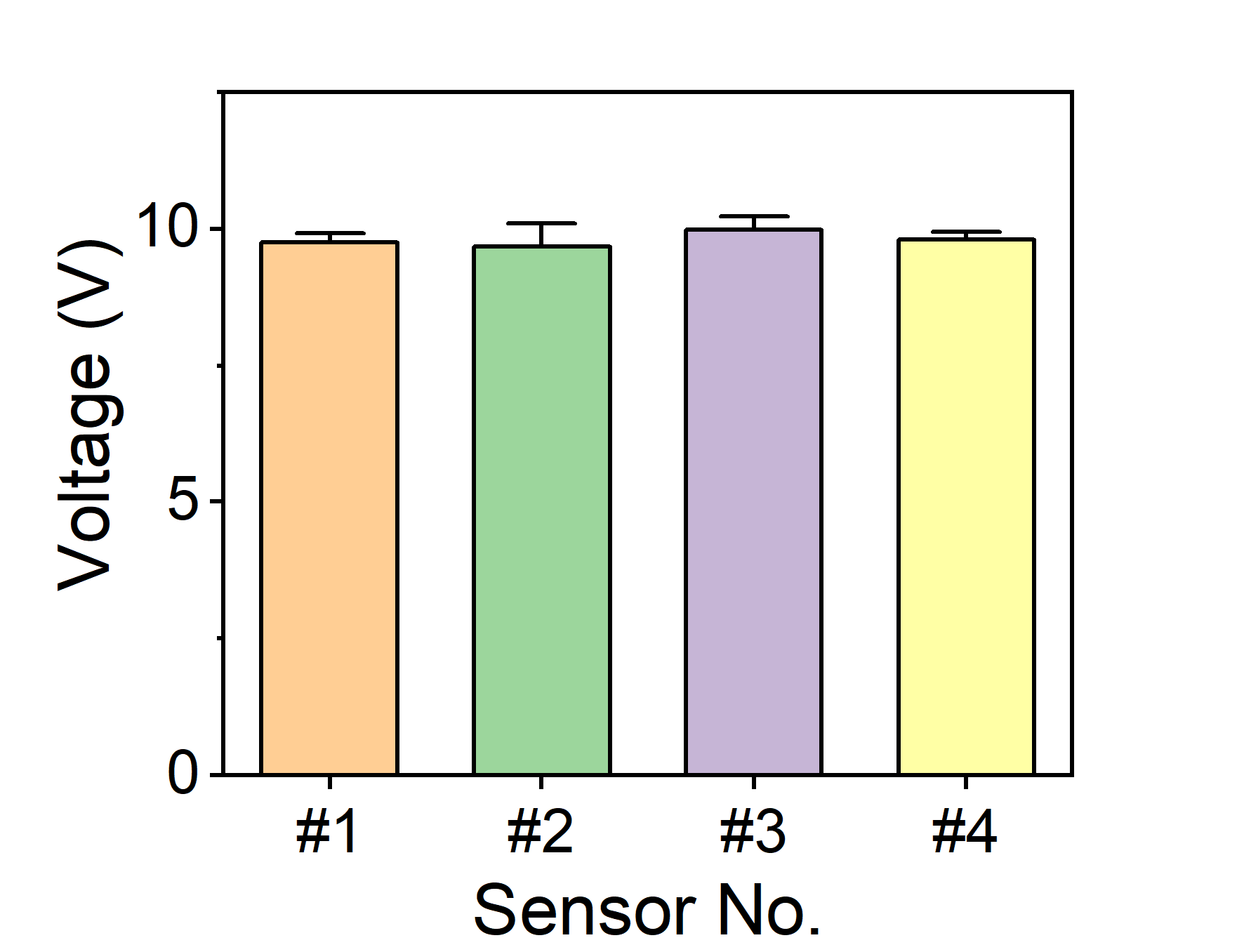


Figure S8. Open-circuit voltage measurements for different sensors at a 90-degree bending angle (100 repetitions). The maximum standard deviation of the output amplitude for any single sensor do not exceed 3% relative to the baseline, while the average output difference between sensors is within 2% of the baseline.


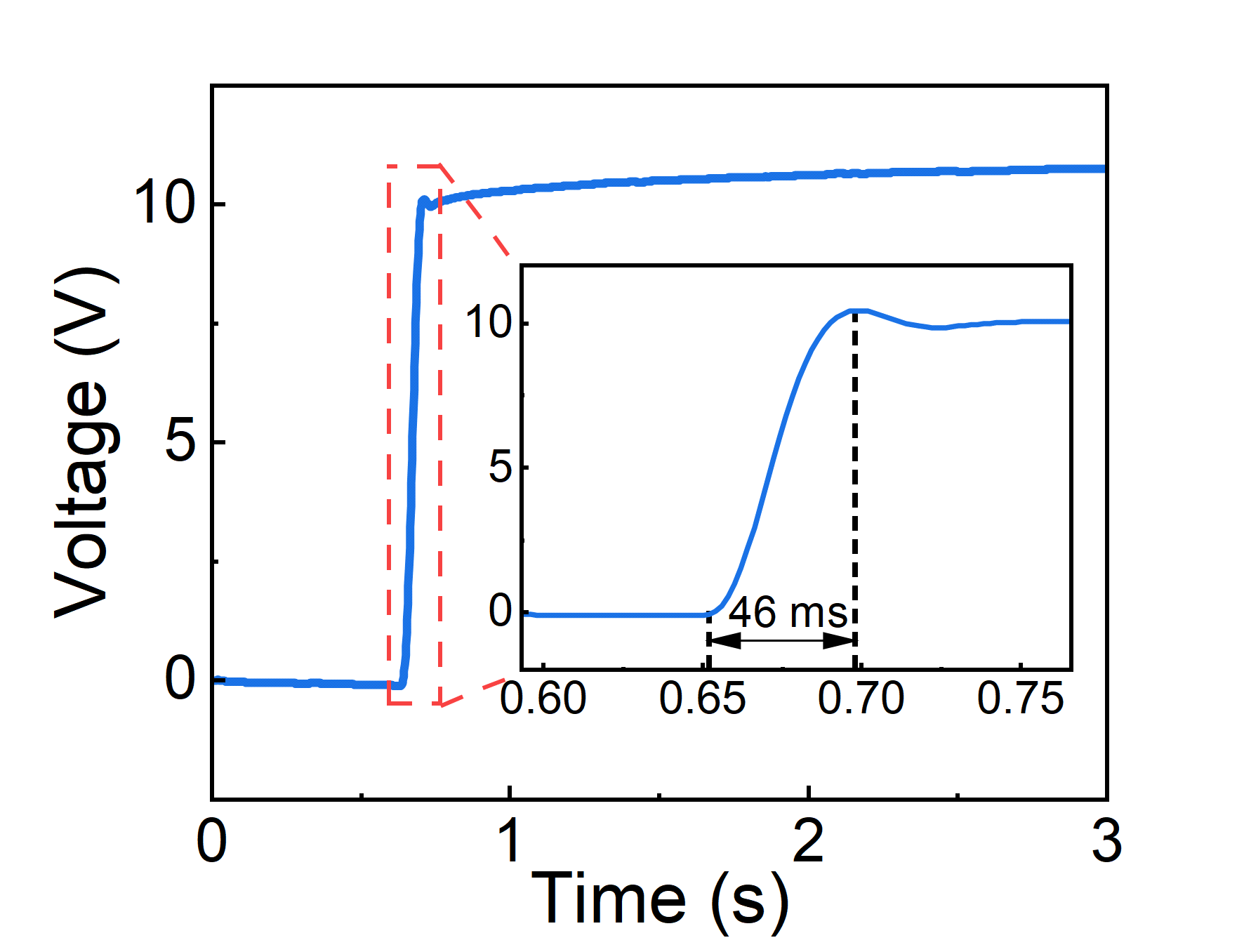


Figure S9. The response curve of the triboelectric sensor. Enlarged: Response time of the sensor is 46 ms.


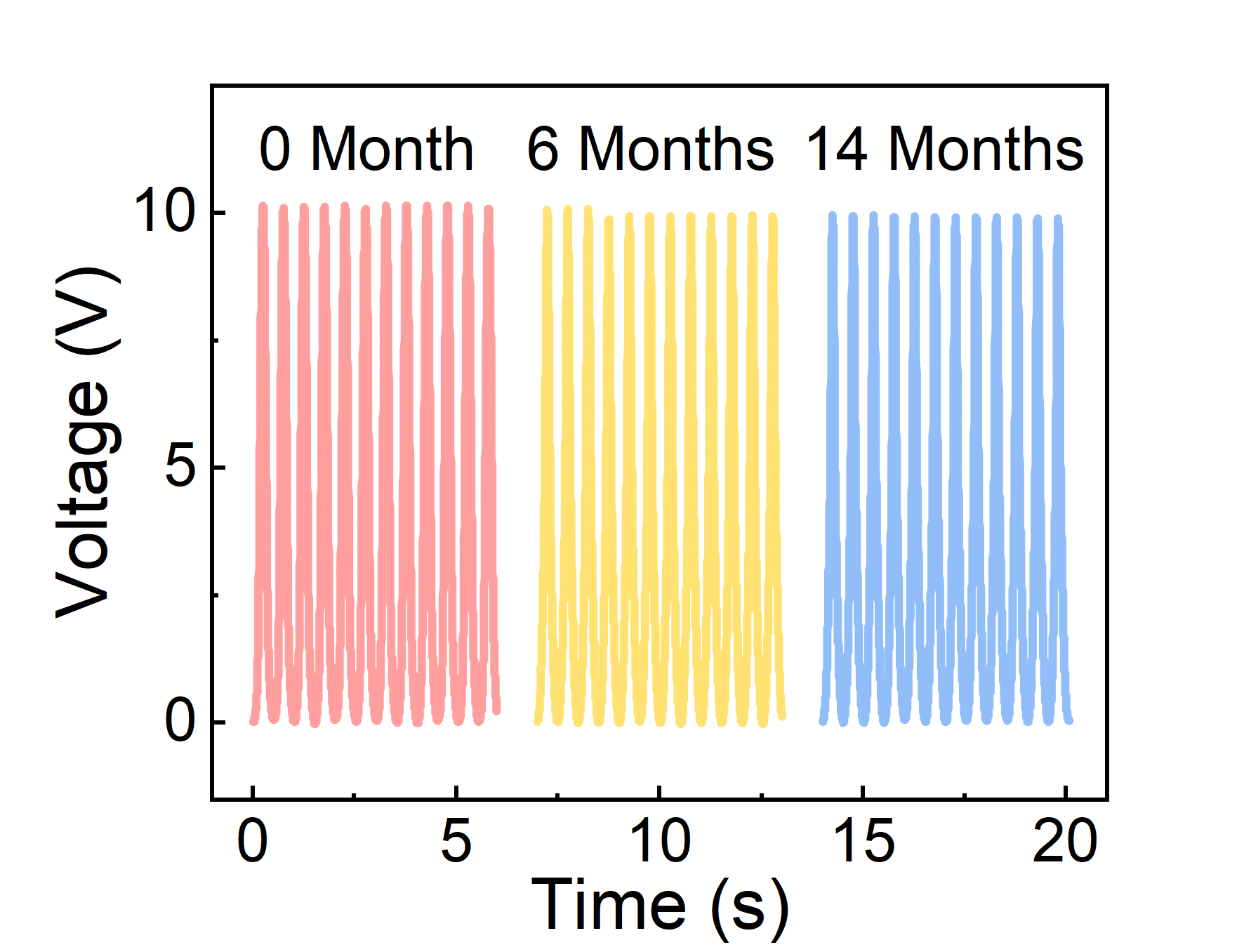


Figure S10. No significant degradation of the sensor's output signal over a period of 14 months.


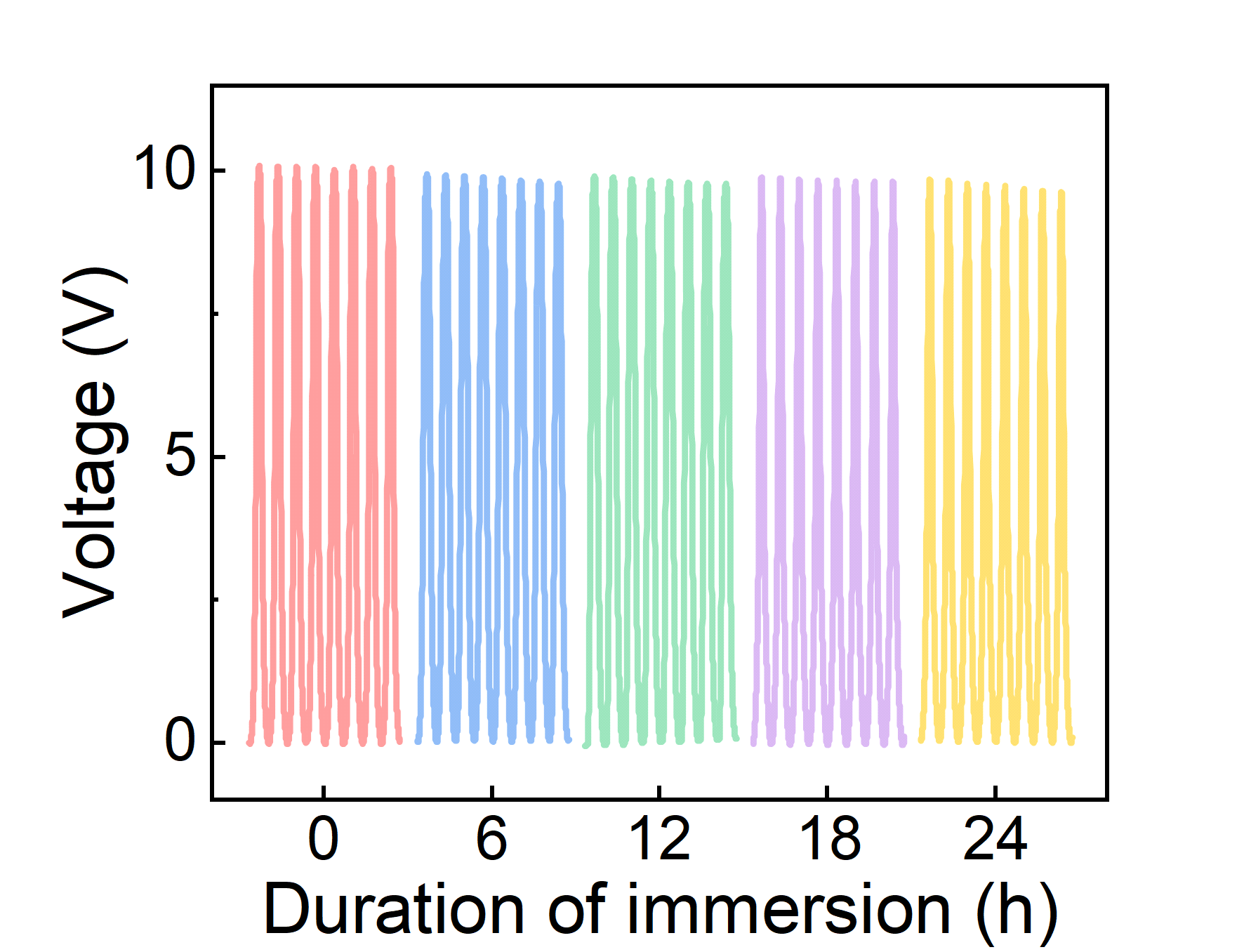


Figure S11. The output signals of the sensor during a 24-hour immersion period.

**
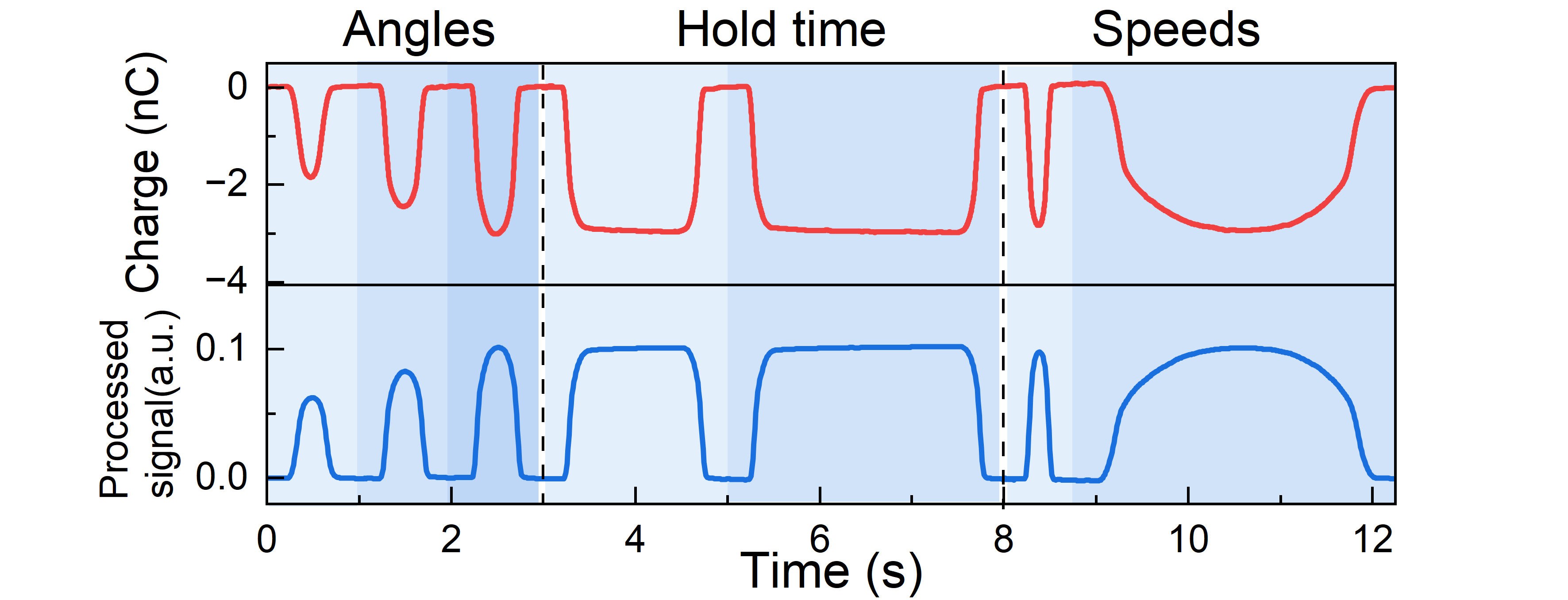
**

Figure S12. The comparison of output charge and PCB acquisition signals at different angles, hold time and speeds during sensor bending.


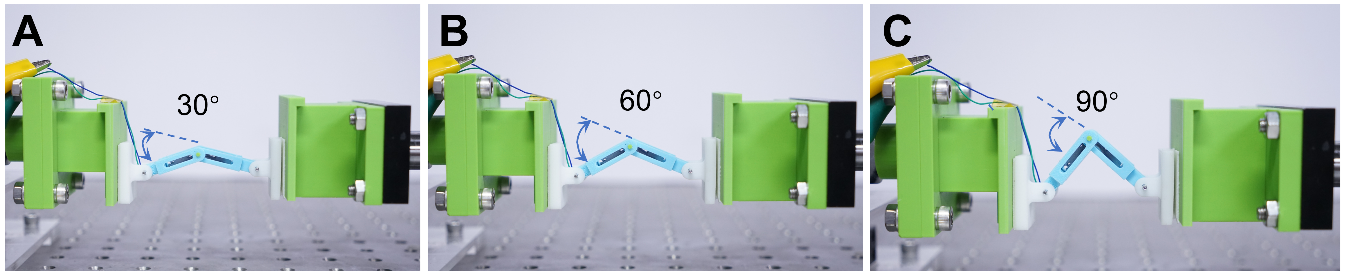


Figure S13. Photographs of the sensor at various bending angles: (A) 30°, (B) 60°, and (C) 90°.


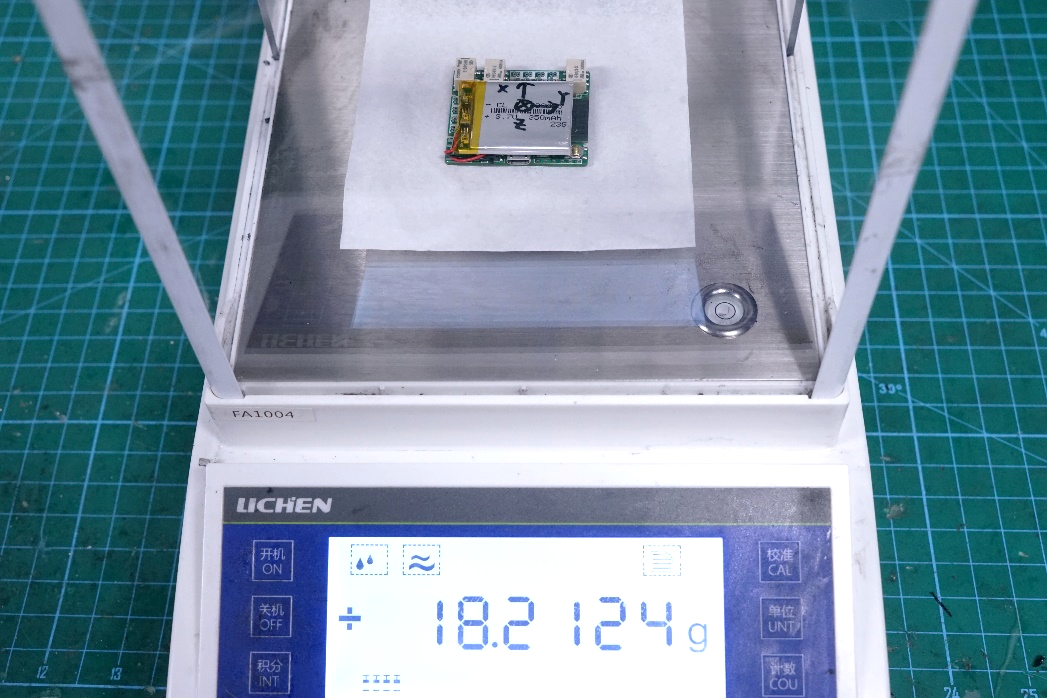


Figure S14. Photograph of the customized PCB being weighed on an electronic scale at only 18.2 g (including the battery).


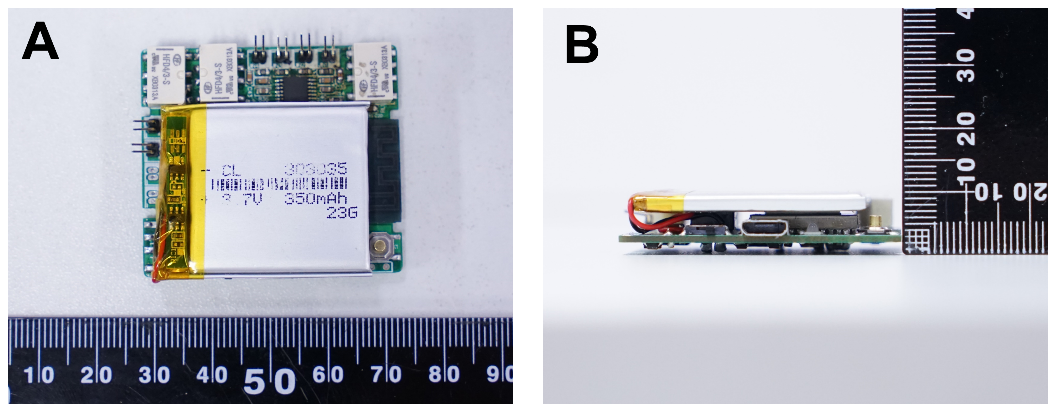


Figure S15. Physical diagrams of the customized PCB. (A) Top view. (B) Front view.

**
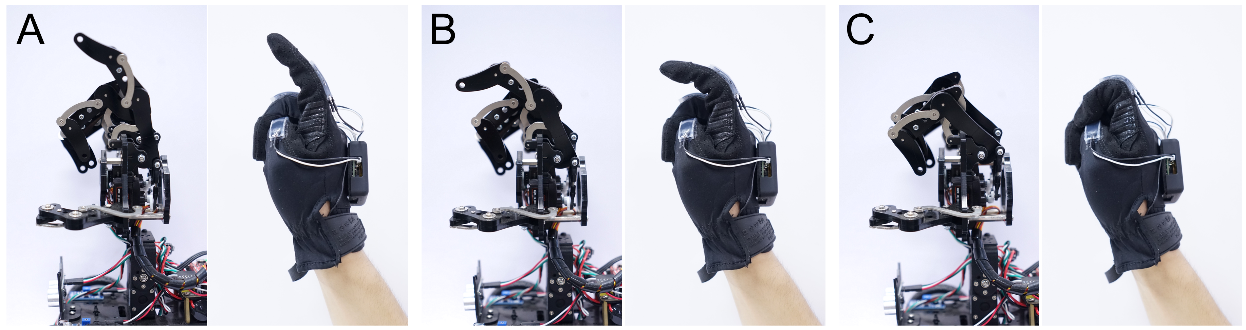
**

Figure S16. Response of the robotic finger to different bending angles of the human finger. (A) Small-angle bending. (B) Medium-angle bending. (C) Large-angle bending.


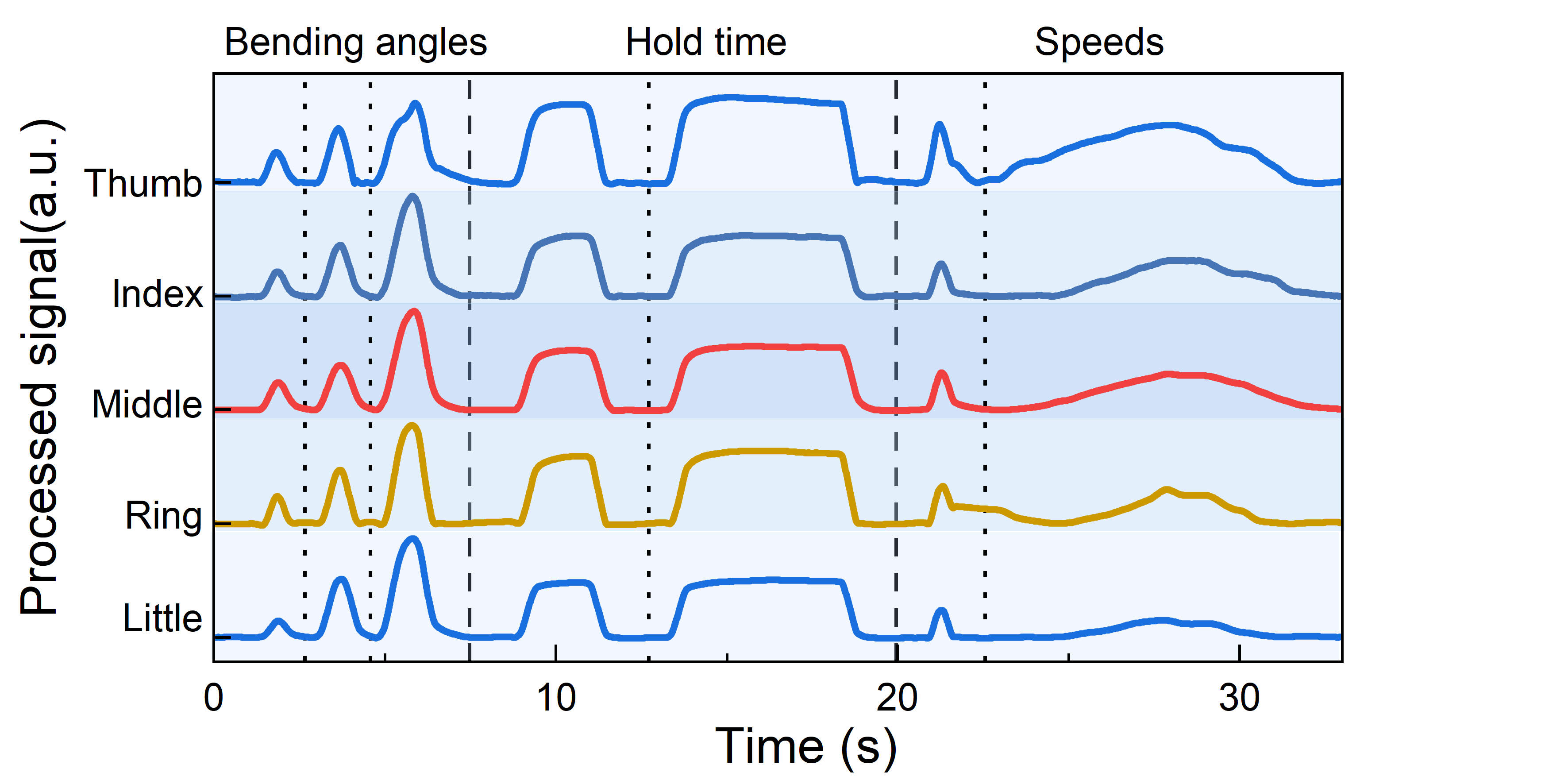


Figure S17. Output signals from triboelectric sensors controlling the movement of the robotic hand at three different bending angles, two different hold times and two different bending speeds.


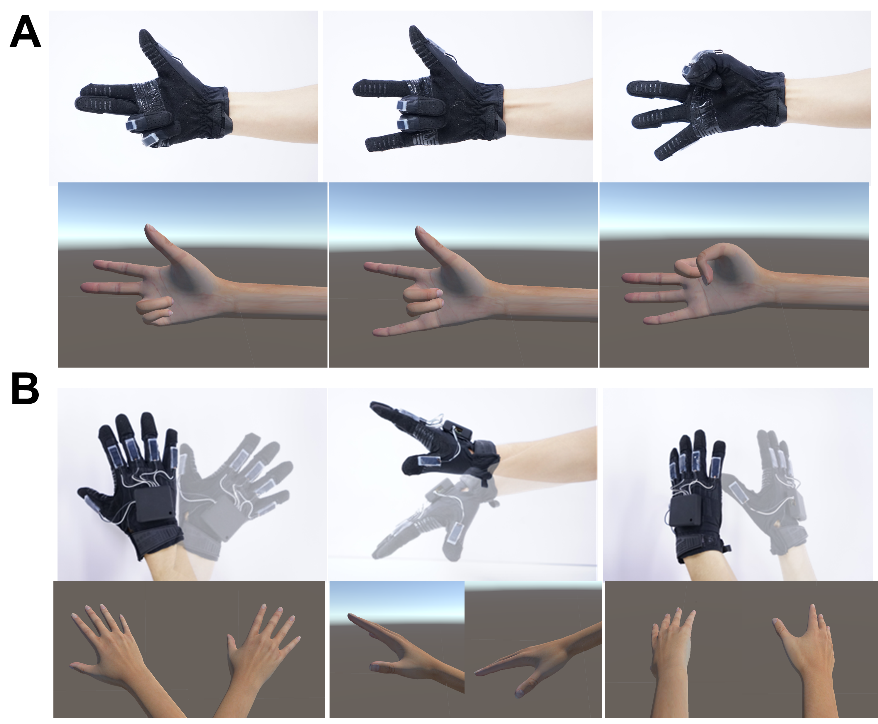


Figure S18. The TI-Glove synchronizes hand movements in the virtual world through (A) different finger bending and (B) different hand orientations.


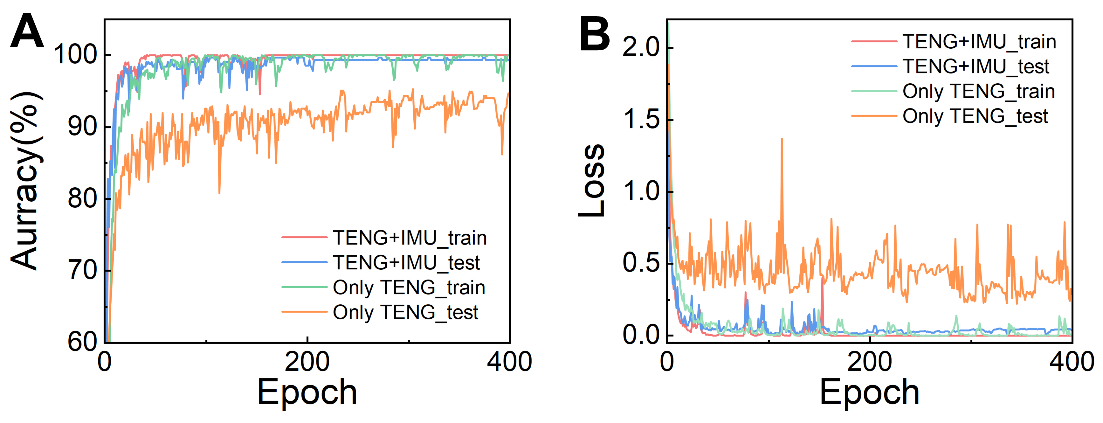


Figure S19. The (A) accuracy curves and (B) loss curves of the model during training and testing.

Table S1. Comparison of glove-based intelligent systems.

| Ref | Finger Bending | | | Hand Posture | Gesture/object Recognition | Wireless Transmission | Fully Portable System |
| --- | --- | --- | --- | --- | --- | --- | --- |
|  | Principle | Continuous  Tracking | Self-powered |  |  |  |  |
| [14] | Piezoresistive | √ |  | √ | √ | √ | √ |
| [15] | Piezoresistive | √ |  |  |  | √ | √ |
| [16] | Capacitive | √ |  |  | √ |  |  |
| [18] | Fiber-optic | √ |  |  |  | √ | √ |
| [25] | Triboelectric |  | √ |  | √ | √ |  |
| [27] | Triboelectric |  | √ |  | √ | √ | √ |
| [28] | Triboelectric | √ | √ |  | √ | √ | √ |
| [31] | Triboelectric | √ | √ |  | √ |  |  |
| [32] | Triboelectric |  | √ |  |  |  |  |
| [41] | Triboelectric | √ | √ |  | √ |  |  |
| This work | Triboelectric | √ | √ | √ | √ | √ | √ |

Table S2. Performance comparison of various triboelectric signal measurement schemes.

| Ref | Signal Type | Measurement Platform | Continuous Tracking | Data Transmission | Sampling Rate (Hz) | Channel Number | Overall weight (g) |
| --- | --- | --- | --- | --- | --- | --- | --- |
| [28] | Voltage Integration | Customized PCB | Yes | Wireless | 100 | 5 | N/A |
| [24] | Voltage | Electrometer | Yes | Cable | 500 | 1 | 4600 |
| [26] | Voltage | Collection card | No | Cable | 500 | 4 | >170 |
| [22] | Voltage | Oscilloscope | No | Cable | N/A | 3 | 9800 |
| [27] | Voltage | Customized PCB | No | Wireless | 250 | 5 | 17.8 |
| This work | Charge Quantity | Customized PCB | Yes | Wireless | 500 | 5 | 18.2 |

Table S3. Power consumption of each unit in the system.

|  | Signal processing unit | ADC | IMU | Wi-Fi module | Overall |
| --- | --- | --- | --- | --- | --- |
| Unit Number | 2 | 2 | 1 | 1 |  |
| Power consumption (each unit) | 50 μW | 12 mW | 9.25 mW | 0.42 W (Active)  0.79 mW (Sleep) | 0.48 W (Active) |

Table S4. The parameters for one-dimensional convolutional neural network (1D-CNN).

| No. | Layer Type | Filters | Kernel Size | Stride | Padding | Input Size | Output Size |
| --- | --- | --- | --- | --- | --- | --- | --- |
| 1 | Convolution 1 | 16 | 7 | 1 | same | (batch_size, 8, 400) | (batch_size, 16, 400) |
| 2 | Max-pooling 1 |  | 3 | 2 | same | (batch_size, 16, 400) | (batch_size, 16, 200) |
| 3 | Convolution 2 | 32 | 9 | 1 | same | (batch_size, 16, 200) | (batch_size, 32, 200) |
| 4 | Max-pooling 2 |  | 3 | 2 | same | (batch_size, 32, 200) | (batch_size, 32, 100) |
| 5 | Convolution 3 | 64 | 11 | 1 | same | (batch_size, 32, 100) | (batch_size, 64, 100) |
| 6 | Avg-pooling |  | 3 | 2 | same | (batch_size, 64, 100) | (batch_size, 64, 50) |
| 7 | Convolution 4 | 128 | 13 | 1 | same | (batch_size, 64, 50) | (batch_size, 128, 50) |
| 8 | Flatten |  |  |  |  | (batch_size, 128, 50) | (batch_size, 6400) |
| 9 | Dense 1 |  |  |  |  | (batch_size, 6400) | (batch_size, 128) |
| 10 | Dense 2 |  |  |  |  | (batch_size, 128) | (batch_size, 10) |

Table S5. Benchmark table for multiple machine learning (ML) architectures.

| Models | Accuracy | F1 Score | Training Duration | Inference Time |
| --- | --- | --- | --- | --- |
| RF | 0.9267 | 0.9289 | 2.2 s | 2.20 ms |
| SVM | 0.9133 | 0.9157 | 0.1 s | 0.24 ms |
| RNN | 0.9533 | 0.9518 | 16.4 s | 0.020 ms |
| LSTM | 0.9867 | 0.9861 | 145.6 s | 0.016 ms |
| CNN | 0.9938 | 0.9936 | 33.1 s | 0.015 ms |

Movie S1.

Robotic gesture control.

Movie S2.

Multi-dimensional robotic finger control.

Movie S3.

Integrated demonstration of robotic control.

Movie S4.

Demonstration of virtual reality interaction.

Movie S5.

Demonstration of light adjustment.

Movie S6.

Demonstration of intuitive interface control.

Movie S7.

Demonstration of sign language recognition.
